# Depression and quality of life in Tunisian institutionalized elderly subjects

**DOI:** 10.1192/j.eurpsy.2022.1675

**Published:** 2022-09-01

**Authors:** M. Jabeur, L. Gassab, F. Hamdane, B. Amemou, F. Zaafrane, L. Gaha

**Affiliations:** Research laboratory LR 05 ES 10 “Vulnerability to Psychotic Disorders”, Faculty of medicine, University of Monastir, Psychiatry Department, University Hospital Of Monastir, Monastir, Tunisia

**Keywords:** elderly subject, institution, Depression, Quality of Life

## Abstract

**Introduction:**

Depression in the elderly is common and closely interrelated with the deterioration of the quality of life, especially in the institutionalized elderly.

**Objectives:**

In this work, we propose to determine the prevalence of depression in the elderly in institution, to assess their quality of life and to evaluate the correlations between depression and the quality of life.

**Methods:**

Our study concerned 30 elderly subjects institutionalized at the retirement home(Sousse, Tunisia). Three validated Arabic version scales were used: The 30-item GDS (Geriatric Depression Scale), the MMSE (Mini Mental State Examination) and the SF36 (assessing the quality of life).

**Results:**

The mean age of our population was 75±7.3 years, the sex ratio was 1.73. The prevalence of depression was 37%. The elderly had a cognitive impairment in 16.7%. The mean global SF36 score were 11.2, attesting an altered quality of life in all our subjects: the mental component (9.43) were more altered than the physical one (13.03). No correlation between depression and quality of life was found. Depression was significantly correlated with the presence of a medical history (p=0.05). Depression had a negative and statistically significant correlation with the physical score of SF36 (r=-0.41, p=0.02) and tended towards significance for the “general health” dimension of SF36 (r=-0.32, p=0.08).

**Conclusions:**

Our study shows a high frequency of depression in the institutionalized elderly as well as a deterioration in their quality of life. Depression is strongly linked to deterioration in physical condition.Our results underline the influence of somatic diseases as a major risk factor for depression in the elderly.

**Disclosure:**

No significant relationships.

